# Amyloidosis, Inflammation, and Oxidative Stress in the Heart of an Alkaptonuric Patient

**DOI:** 10.1155/2014/258471

**Published:** 2014-04-28

**Authors:** Lia Millucci, Lorenzo Ghezzi, Eugenio Paccagnini, Giovanna Giorgetti, Cecilia Viti, Daniela Braconi, Marcella Laschi, Michela Geminiani, Patrizia Soldani, Pietro Lupetti, Maurizio Orlandini, Chiara Benvenuti, Federico Perfetto, Adriano Spreafico, Giulia Bernardini, Annalisa Santucci

**Affiliations:** ^1^Dipartimento di Biotecnologie, Chimica e Farmacia, Università degli Studi di Siena, Via Fiorentina 1, 53100 Siena, Italy; ^2^Dipartimento di Scienze della Vita, Università degli Studi di Siena, Via A. Moro, 53100 Siena, Italy; ^3^Dipartimento di Scienze Fisiche, della Terra e dell'Ambiente, Università degli Studi di Siena, Via Laterina, 53100 Siena, Italy; ^4^Medicina e Riattivazione Cardiologica DAI Cuore e Vasi, AOU Careggi, 50139 Firenze, Italy; ^5^Centro di Riferimento Regionale per lo Studio dell'Amiloidosi, Dipartimento di Medicina Sperimentale e Clinica, Università degli Studi di Firenze, Viale Pieraccini 18, 50139 Firenze, Italy; ^6^Immunoematologia Trasfusionale, Azienda Ospedaliera Universitaria Senese, Policlinico Le Scotte, 53100 Siena, Italy

## Abstract

*Background*. Alkaptonuria, a rare autosomal recessive metabolic disorder caused by deficiency in homogentisate 1,2-dioxygenase activity, leads to accumulation of oxidised homogentisic acid in cartilage and collagenous structures present in all organs and tissues, especially joints and heart, causing a pigmentation called ochronosis. A secondary amyloidosis is associated with AKU. Here we report a study of an aortic valve from an AKU patient. *Results*. Congo Red birefringence, Th-T fluorescence, and biochemical assays demonstrated the presence of SAA-amyloid deposits in AKU stenotic aortic valve. Light and electron microscopy assessed the colocalization of ochronotic pigment and SAA-amyloid, the presence of calcified areas in the valve. Immunofluorescence detected lipid peroxidation of the tissue and lymphocyte/macrophage infiltration causing inflammation. High SAA plasma levels and proinflammatory cytokines levels comparable to those from rheumatoid arthritis patients were found in AKU patient. *Conclusions*. SAA-amyloidosis was present in the aortic valve from an AKU patient and colocalized with ochronotic pigment as well as with tissue calcification, lipid oxidation, macrophages infiltration, cell death, and tissue degeneration. A local *HGD* expression in human cardiac tissue has also been ascertained suggesting a consequent local production of ochronotic pigment in AKU heart.

## 1. Introduction

Alkaptonuria (AKU; MIM number 203500) is an ultrarare (1 : 250.000–1.000.000 incidence) autosomal recessive inborn error of catabolism of the aromatic amino acids due to a deficient activity of the enzyme homogentisate 1,2-dioxygenase (HGD) leading to the accumulation of homogentisic acid (2,5-dihydroxyphenylacetic acid; HGA). HGA-oxidized derivative benzoquinone acetic acid (BQA) forms melanin-based polymers, deposited in the connective tissue of various organs, causing a pigmentation known as “ochronosis,” leading to dramatic tissue degeneration. A severe form of arthropathy is the most common clinical presentation of AKU but patients often suffer also from cardiovascular disease (frequent cause of death) and kidney disease and though organs may be affected.

As many as 40% of AKU patients experience cardiovascular symptoms starting from the third-fourth decade of life. [1].

Alkaptonuric ochronosis can be treated symptomatically during the early stages, whereas for end stages total joint and heart valve replacements may be required. Currently, there is no appropriate therapy for AKU, although a phase II clinical trial with nitisinone is in progress.

There have been increasing reports of cardiovascular ochronosis [1–8]. Ochronosis is associated with aortic valve stenosis but mitral and pulmonary valves can be affected as well. Numerous case reports have suggested that cardiovascular calcification and stenosis may be associated with pigment deposition in the aortic and mitral valves, endocardium, pericardium, aortic intima, and coronary arteries. Butany and colleagues [2] reported that pigmentation leads to an inflammatory reaction and to progressive valve dysfunction. Usually, pigmentation is associated with age-related valvular calcification [9, 10]. Although most AKU cases presented aortic stenosis, Yoshikai and colleagues [8] reported a case of aortic valve regurgitation in alkaptonuria.

We reported that AKU is a secondary serum amyloid A-(SAA-) based amyloidosis and amyloid deposits were revealed in cartilage, synovia, and bone, also due to a local* HGD *expression in the osteoarticular system [11, 12]. By means of our* in vitro* cell and tissue AKU models we also proved that HGA is responsible for pigment and SAA and amyloid production and a colocalization of ochronotic pigment and SAA-amyloid was also reported [9, 11–19].

In the present work, we show that SAA-amyloidosis was present in the aortic valve from an AKU patient. SAA-amyloid colocalized with ochronotic pigment as well as with tissue calcification, lipid oxidation, lymphocytes/macrophages infiltration, cell death, and tissue degeneration. A local* HGD* expression in human cardiac tissue has also been ascertained suggesting a consequent local production of ochronotic pigment in AKU heart.

## 2. Materials and Methods

The whole study was conducted following the approval of Siena University Hospital Ethics Committee and has therefore been performed in accordance with the ethical standards laid down in the 1975 Declaration of Helsinki and its later amendments. The patient gave a written informed consent prior to inclusion in the study.

All reagents were from Sigma-Aldrich (St. Louis, MO), if not differently specified.

### 2.1. AKU Patient

Alkaptonuric specimen was obtained from a 65-year-old woman who underwent biologic aortic valve replacement and had been previously diagnosed for AKU-related secondary amyloidosis as SAA-amyloid had been detected in her cartilage and synovia [16, 20].

### 2.2. Histochemical Analysis

Valve sections of 5 to 7 *μ*m were rinsed in PBS, stained with eosin, counterstained with Gill's hematoxylin, dehydrated in ethanol and xylene, and mounted in Eukitt (O. Kindler Gimbtt Co.). Images were obtained using a Zeiss Axio Lab.A1 microscope and captured using AxioVision Rel.4.8 Image software. Melanin-like ochronotic pigment was revealed by Fontana-Masson staining.

### 2.3. Scanning Electron Microscopy (SEM)

SEM observations and chemical microanalysis were carried out using a Philips XL30 device operated at 20 kV and equipped with an EDAX energy dispersive (EDS) X-ray. The volume of sample analyzed with EDAX, at the actual operating condition, was estimated to have a diameter of ca. 3 *μ*m; hence areas smaller than ca. 5 *μ*m will have chemical analyses mixed with adjacent phases. As a first step the fragment was analyzed without carbon coating to check the presence of C in the sample itself. Once verified that C was a component of the ochronotic pigment, the fragment was carbon coated in order to obtain good quality images under SEM.

### 2.4. Transmission Electron Microscopy (TEM)

AKU aortic valve was fixed in 2.5% glutaraldehyde in 0.1 M cacodylate buffer (CB) pH 7.2 for 3 h at 4°C. After rinsing in CB, sample was postfixed in 1% osmium tetroxide in CB for 2 h at 4°C, dehydrated in a graded series of ethanol, and embedded in a mixture of Epon Araldite resins. Thin sections, obtained with a Reichert ultramicrotome, were stained with uranyl acetate and lead citrate and observed with a TEM FeiTecnai G2 spirit at 80 Kv.

### 2.5. Congo Red (CR) Staining

A version of Romhányi's original CR staining method [21] modified according to Bély and Apáthy [22] was adopted. Sections of 5 *μ*m thickness of fresh aortic valve specimen were fixed in cooled 96% ethanol 10 min, rinsed in distilled water, incubated in 1% CR for 40 min, washed in water, incubated 10 s in 1 mL 1% sodium hydroxide in 100 mL of 50% ethanol, incubated 30 s in Mayer's hematoxylin, sequentially washed in 50%, 75%, and 95% ethanol, mounted, and observed under a polarized light microscope (Zeiss Axio Lab.A1, Arese, Milano).

### 2.6. Fluorescence Microscopy

Frozen AKU aortic valve was cut in 5 *μ*m slices and used for immunofluorescence staining with anti-SAA. Additional immunofluorescence assays were performed using anti-4-hydroxy-2-nonenal (4-HNE) antibody (Percipio Biosciences, Inc., USA).

### 2.7. Western Blotting

Tissue sample from human aortic valve was powdered under liquid nitrogen using a pestle and mortar and then lysed in sample lysis buffer (7 M urea, 2 M thiourea, 100 mM DTT, 4% CHAPS, 2% ASB-14, 1 mM Na3VO4, 1 mM NaF, 1% Protease Inhibitors). Samples were left for 15 min in ice, vortexing every 5 min, and centrifuged at 2500 ×g for 20 min. Total protein content of lysate was determined using a Bio-Rad protein assay. Twenty micrograms of sample were resolved by SDS-PAGE, electrotransferred onto nitrocellulose membranes, and probed with Anti-HGD or Anti-GAPDH antibodies, followed by Anti-Rabbit HRP-conjugated antibody. Detection was obtained by ImmunoStarHRP (Bio-Rad, Segrate, Milan, Italy); images were acquired using ImageScannerIII (GE Healthcare, Milan, Italy) and analyzed by ImageQuantTL (GE).

### 2.8. Biochemical Assay

Plasma SAA and serum amyloid P (SAP) levels in AKU patient were measured by ELISA (Invitrogen-Life Technologies, Carlsbad, CA). A panel of proinflammatory cytokines was evaluated by Bioplex (Bio-Rad, Milan, Italy) in AKU patient's plasma and in a pool of 15 plasma from rheumatoid arthritis patients.

### 2.9. Statistical Analysis

Student's *t*-test was used when appropriate. Two-tailed analysis with *P* value lower than 0.05 was considered significant. Correlation analysis was performed using Pearson's correlation.

## 3. Results

### 3.1. Macroscopic and Microscopic Observation of AKU Aortic Valve

**Figure 1:**
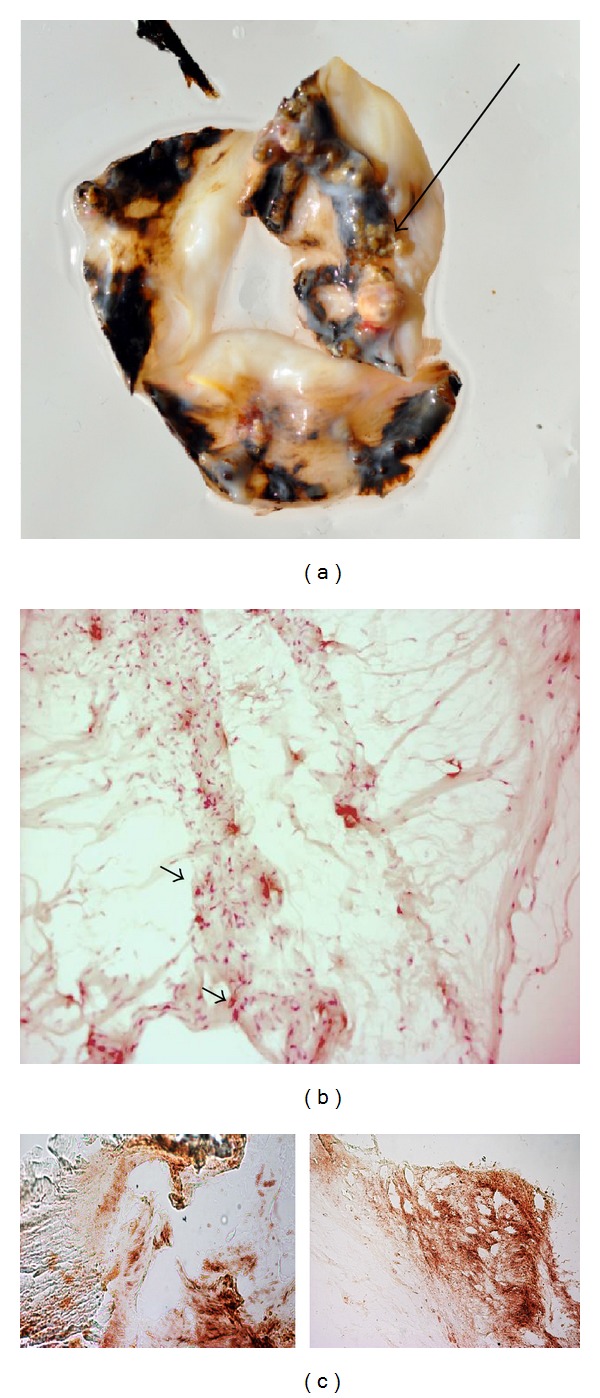
(a) Ochronotic pigmentation on the surface of the valve was visible. A thickening of the central areas of the cusps with nodule-like structures is visible (arrow). (b) Light microscopy (hematoxylin and eosin staining) examinations of AKU aortic valve with intense inflammatory cell infiltrate that includes large cells macroscopically corresponding to macrophages (arrows). Magnification 20x. (c) Melanin-like ochronotic pigment was particularly detected by Fontana-Masson staining. Magnification 10x.

Ochronotic pigment with sclerotic calcification in the cusps was visible in the valve (Figure 1(a)). The gross surface morphology of the aortic leaflets was very rough and irregular and the endothelium showed clear tearing. Calcific deposits in the valve tissues always occurred in the vicinity of the endothelial defects. Large deposits of extracellular ochronotic pigment were associated with areas of valvular calcification. Intracellular and extracellular deposits of ochronotic pigment suggested it could function as a stimulus for dystrophic calcification. Light microscopy observation showed various degrees of pathological alterations and severe calcification involving surface endothelium, underlying basement membranes, and deeper layers of interweaving networks of collagen fiber bundles in the pars fibrosa of the valve tissues as well as cellular swelling indicative of degenerative lesions (Figure 1(b)). The ochronotic pigmentation observed using HE stained sections was also confirmed using Fontana-Masson staining (Figure 1(c)). The argentaffin reaction due to Fontana-Masson staining was observed in the aortic valve leaflets, which confirmed the presence of massive melanin-like pigment in AKU aortic valve.

### 3.2. SEM Observation

**Figure 2:**
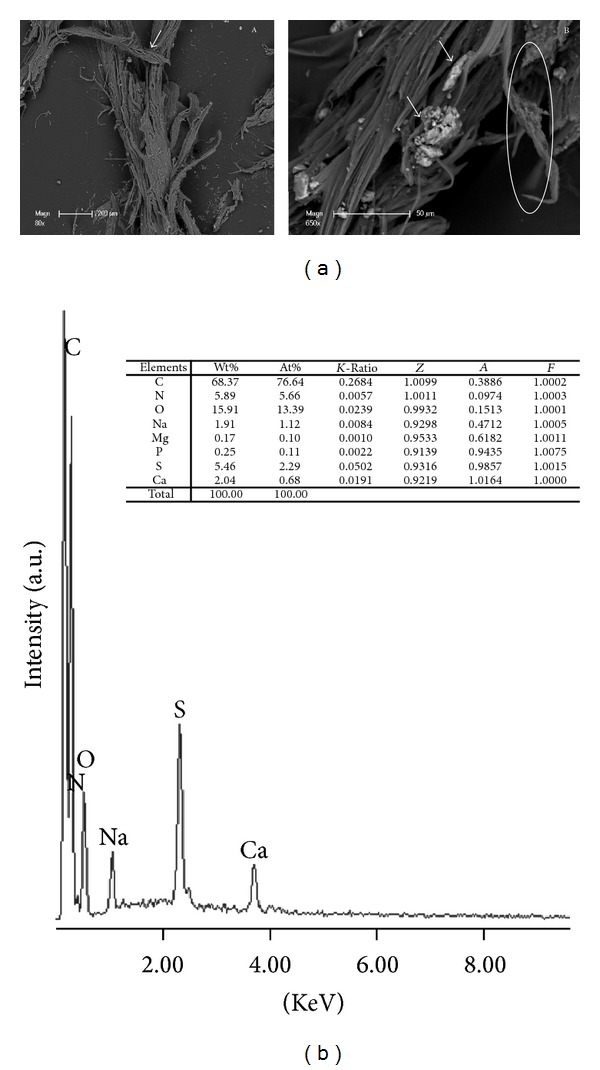
(a)* SEM images of AKU aortic valve*. (A) Ultrastructural features of typical filiform (arrow) Lambl's excrescences and massive desquamation of superficial layers are reported. (B) Tissue fibers adjacent to calcium deposits interconnecting bundles of collagen and evident defects on the valve surface disrupting the endothelial cover are shown. Arrows indicate hydroxyapatite concretions. Circles highlight calcium deposits interconnecting bundles of fibrous tissue. (b)* EDS spectra of pigmented area of AKU aortic valve*. Table reports ochronotic pigment element composition.

SEM and chemical microanalysis were carried out, to investigate the tissue external surface and inner microstructure, respectively. We collected both back scattered (BSE) and secondary electron (SE) images. We found significant alterations throughout the structure of AKU aortic valve. Loose binding of endothelial cells together with a loss of the endothelial layer over more or less widespread areas of the leaflets was evident (Figure 2(a)AB). Also, diffuse pigment deposition seemed to result in the desquamation of the endothelial coverage (Figure 2(a)). The valve presented a filamentous dark surface embedding small (10–20 *μ*m) white concretions (Figure 2(a)B). Images showed prominent villous excrescences having a lamellar appearance (Figure 2(a)). Ultrastructural features of typical filiform and lamellar Lambl's excrescences provided evidence that the alteration of endothelial integrity plays a contributory role in calcific degeneration in the aortic valve (Figure 2(a)). Fibres adjacent to calcium deposits interconnecting bundles of collagen on the valve surface were shown (Figure 2(a)B). The concretions enclosed within hollow spaces were grouped by interconnecting bundles of deteriorated collagen fibers (Figure 2(a)B).

### 3.3. EDS Microanalyses

To get light on the composition of ochronotic pigment we performed EDS microanalyses on the dark surface of the valve (Figure 2(b)). Results were very reproducible and revealed that pigmented areas were composed by C, O, N, S, Na, and variable amount of Ca. Sulphur is an element never reported before in cardiac valve tissue, and its constant presence in the pigment is probably due to the ability of HGA oxidized to BQA, to form adducts with protein thiols [6]. The white concretions (Figure 2(a)B) contained P and Ca that indicated the presence of hydroxyapatite. Such a mineral phase indicated that in AKU aortic valve a process of endochondral ossification like in bone was occurring, as also evidenced by SEM images with collagen appearing as delicate bundles of fibres with a random orientation and exhibiting like bone concretions (Figure 2(a)B). Hence, it is an active process rather than passive calcification.

### 3.4. TEM Observation of Amyloid Deposits

**Figure 3:**
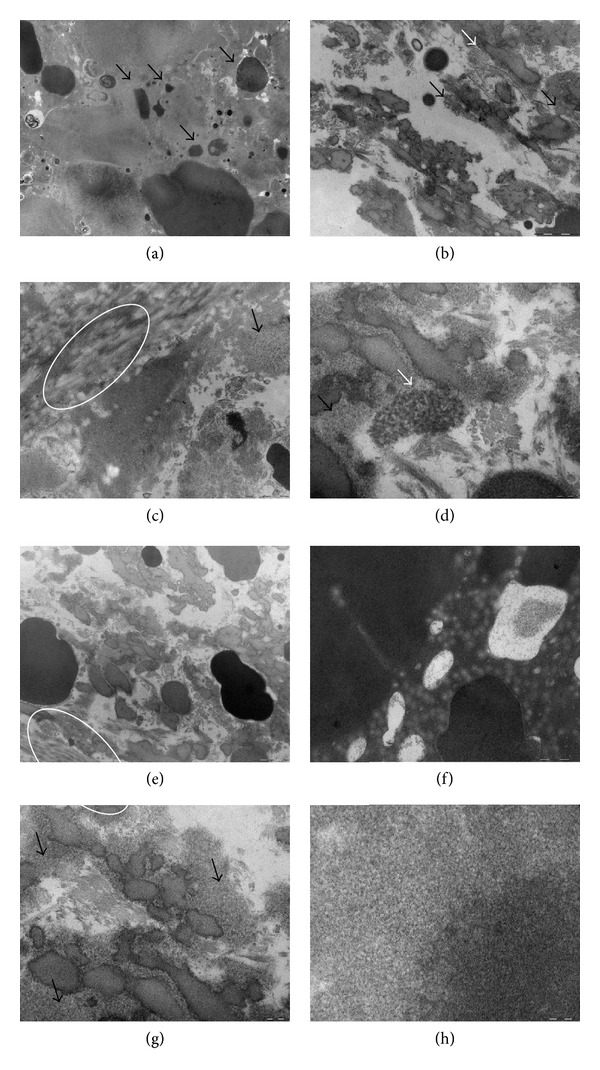
*TEM observation of AKU aortic valve*. (a) Large deposits of extracellular ochronotic pigment associated with areas of valvular calcification (arrows). Bar 2 *μ*m. (b) Pathological alterations and calcification (white arrow) involving surface endothelium with cells disgregation and sprinkled Golgi complex and ER (black arrows). Bar 1 *μ*m. (c) Interweaving networks of collagen fiber bundles (white circle) and aggregates of extracellular ochronotic pigment appeared to be in locations of necrotic cells (black arrow). Bar 1 *μ*m. (d) Intracellular ochronotic pigment (black arrow) and an example of apoptotic cell with condensed chromatin and disrupted mitochondrion (white arrow). Bar 200 nm. (e), (f), and (g) ECM organization is disrupted and ECM trilaminar stratification is lost as evidenced by disorganized and fragmented collagens and elastin fibres strictly interconnected to pigment “squirts” (white circle and arrows); in (f) a necrotic cell is shown with multiple dysmorphic mitochondria. (e) Bar 1 *μ*m; (f) bar 500 nm; (g) bar 200 nm; (h) bundles of amyloid fibrils nearby ochronotic deposits. Bar 100 nm.

**Figure 4:**
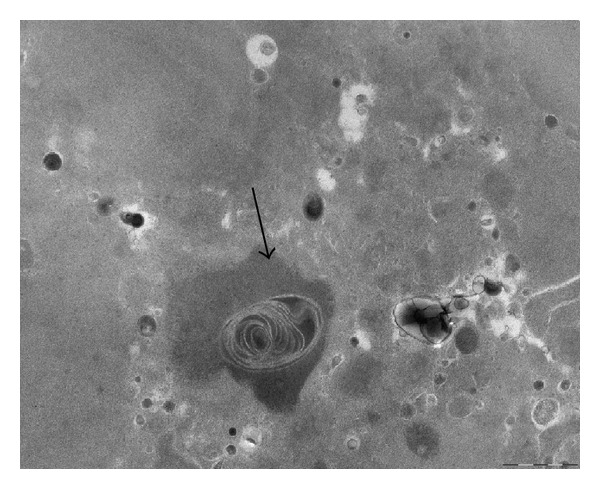
*Myelin figure in AKU aortic valve section. *TEM observation of a myelin figure perfectly superimposing an extended ochronotic pigmented patch (arrow). Bar 1 *μ*m.

The ultrastructural study of the aortic valve revealed intracellular and extracellular deposits of ochronotic pigment (Figures 3(a)–3(d)). Large deposits of extracellular ochronotic pigment were associated with areas of valvular calcification (Figure 3(a)). The specimen consistently showed various degrees of pathological alterations and calcification, involving surface endothelium (Figure 3(b)) and deeper layers of interweaving networks of collagen fibre bundles in the pars fibrosa of the valve tissues (Figure 3(c)). The amount of calcific deposits in the valve tissue increased in proportion to the severity of endothelial damage and gradually decreased from the defective endothelial surface to the deeper layer of collagen tissue. In addition, apoptotic cell death in the valve tissue was massive and probably related to the severity of endothelial injury. Interestingly, aggregates of extracellular ochronotic pigment appeared to be in locations of necrotic cells (Figures 3(c)–3(d)). Moreover, extracellular matrix (ECM) organization was disrupted in AKU valve. Specifically, ECM trilaminar stratification was lost, as evidenced by the presence of disorganized and fragmented collagens and elastin fibres strictly interconnected to ochronotic pigment “squirts” (Figures 3(e), 3(f), and 3(g)). TEM images also showed degenerating or senescent (with many lipid droplets, generalized swelling of organelles, and breaks in the plasma membrane shown in Figures 3(f) and 3(g)) to frankly necrotic (with total absence of plasma membrane and grossly swollen, disintegrating organelles; Figures 3(d)–3(g)) cells. Degenerated cells were randomly distributed throughout all three layers of the leaflet and severely altered or necrotic cells often lay beside those showing little alteration from the normal. The matrix of the valve leaflet connective tissue showed a delicate network of filaments typical of acid mucopolysaccharide material (Figure 3(g)). Occasional cells showed also a slight swelling of the mitochondria (Figure 3(f)), the presence of single lipid droplets (Figures 3(e), 4(g)), or membrane degenerations (Figure 3(a)). Amyloid deposits were found within the AKU valve, and both the colocalization of apoptotic cells with amyloid and their location near ochronotic pigmented centres were observed (Figure 3(h)). We found significant simultaneous occurrence of amyloid deposit and cell degeneration (Figures 3(c)–3(e)), even if the presence of amyloid in the AKU valve was uniform randomly diffused. BQA causes severe cellular injury so that the cell was no longer able to process damaged cellular components [14]. This results in cell death evocating autophagy and is characterized by the accumulation of autophagic vesicles (Figure 3(b)), extensive vacuolization (Figures 3(e) and 3(f)), degradation of membranous cellular components (Figures 3(d), 3(e), and 3(g)) that rearrange in membranous whorls called myelin figure (Figure 4), depletion of organelles, and finally complete disintegration of the cell (Figures 3(f) and 3(g)).

Myelin figures were detected (Figure 4) indicating lipid peroxidation and membrane degeneration. Myelin figures are widely found in a great variety of normal and pathological animal and human somatic and brain tissues [23]. They appear as concentric membranous lamellar formations encapsulating thin bands of cytoplasm and separated by electron lucent and elongated vacuolar spaces (Figure 4). Very intriguing, as shown in Figure 4, in AKU valve myelin figure was perfectly superimposing to an extended pigmented patch, suggesting a strict correlation between cytotoxic action of ochronotic pigment and lipid peroxidation.

### 3.5. Congo Red Staining of Amyloid Deposits

**Figure 5:**
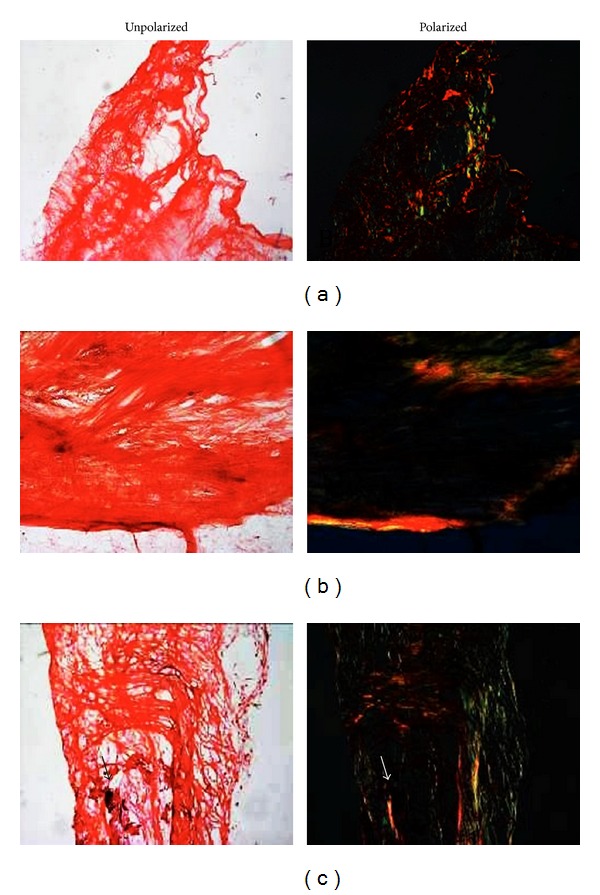
*Congo Red staining of AKU aortic valve amyloid deposits.* Different sections of the valve are shown. Desquamation of endothelial coverage and tissue destruction were evident. Valve tissue was ever stained red (“congophilic”). The pigmented areas (arrows) were also birefringent, indicating overlapping of ochronosis and amyloid. Magnification 40x.

CR staining detected the presence of diffuse amyloid, mainly located in densely sclerotic and poorly vascularised scar tissue (Figure 5(a)), frequently in close proximity to calcific deposits (Figure 5(c)). Amyloid was sometimes present in areas of apparently degenerated fibrous tissue and in some cases (Figure 5(b)) amyloid correlates with diffuse desquamation of superficial tissue layers. Colocalization of amyloid and ochronotic pigment was clearly visible.

### 3.6. SAA Detection

**Figure 6:**
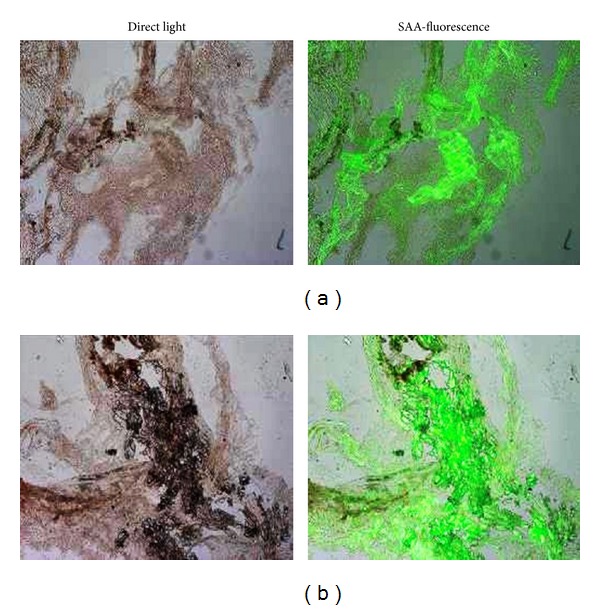
*SAA detection in AKU aortic valve amyloid deposits. *Positive staining for SAA-amyloid was particularly intense in correspondence of ochronotic pigmentation. SAA deposition in AKU aortic valve was detected by immunofluorescence technique. Magnification 20x.

SAA deposition in aortic valve was examined using immunofluorescence techniques. Colocalization of SAA with ochronotic pigmentation was detected in all of the examined sections of the valve (Figure 6).

### 3.7. 4-HNE Detection

**Figure 7:**
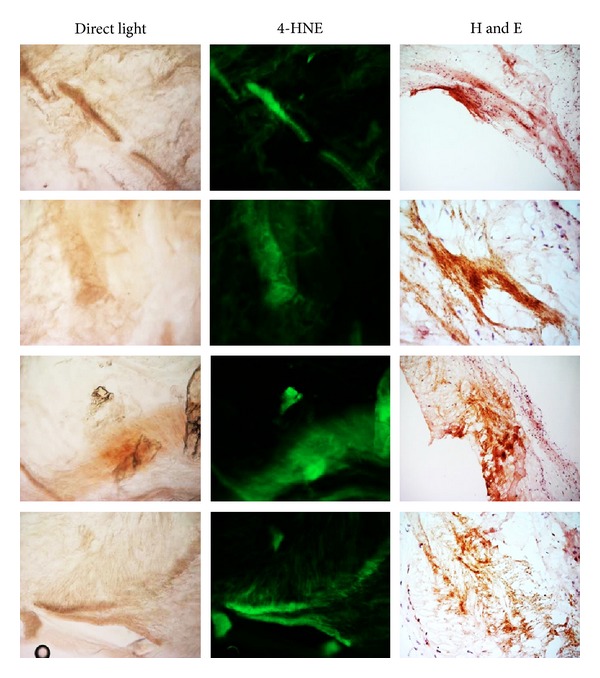
*Immunoreactivity for 4-HNE in AKU aortic valve leaflet. *The presence of 4-HNE in the AKU valve was uniformly diffused and the distribution of 4-HNE-positive area was perfectly superimposing to ochronotic pigmented areas. The presence of lipid peroxidation and ochronotic pigment was found to be strictly related to areas of lymphocytes accumulation. Magnification 20x.

In order to evaluate the presence of major products of lipid peroxidation (LPO), AKU valve sections were incubated with mouse monoclonal anti-4-HNE antibody. LPO was clearly evident in AKU aortic valve (Figure 7). The distribution of 4-HNE-positive areas was perfectly superimposing to pigmented areas, suggesting the association of intraleaflet ochronosis and oxidative stress (Figure 7). AKU valve also contained lymphocytes and macrophages in the subendothelial layer of the fibrosa, in the vicinity of ochronotic deposits, and along the lamina elastic as revealed in hematoxylin and eosin stained slides (Figure 7). An evident colocalization between oxidized lipids, lymphocytes and macrophages accumulation, and ochronotic deposits suggested that LPO might play a role in the disease process.

### 3.8. HGD Expression in Human Aortic Valve

**Figure 8:**
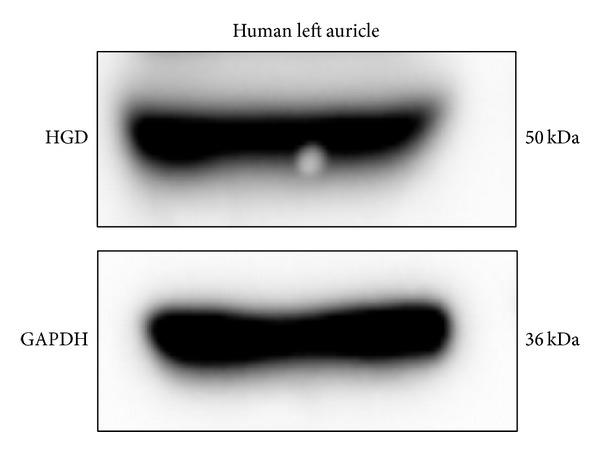
Western blotting with anti-HGD antibody of human aortic valve from a non-AKU patient. Anti-GAPDH antibody as a loading control is shown.

Western blotting with anti-HGD antibodies confirmed the expression of HGD in human aortic valve from a non-AKU patient (Figure 8). This implies a spontaneous ability of AKU heart cells to accumulate HGA and produce ochronotic pigment* in situ*, thus strongly contributing to induction of local ochronosis in AKU aortic valve.

### 3.9. SAA, SAP, and Proinflammatory Cytokines Measurements

**Figure 9:**
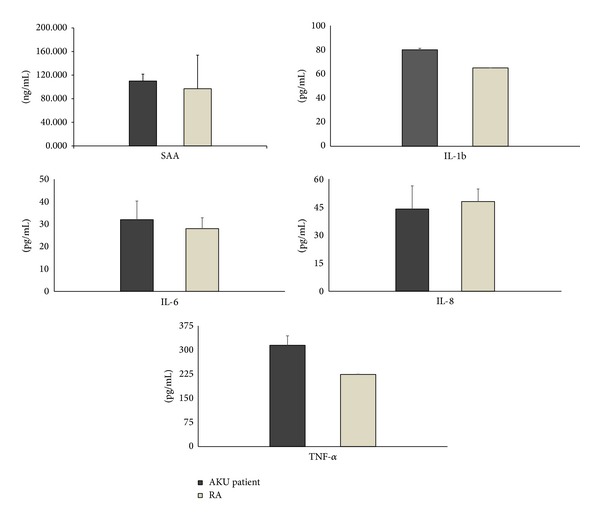
*Evaluation of SAA and the profile of proinflammatory cytokines in the plasma from AKU patient. *SAA plasma levels were evaluated by ELISA while cytokines were assayed by Bioplex. A comparison with levels found in a pool of plasma from 15 rheumatoid arthritis (RA) patients is also reported. Experiments were performed in triplicate; data are presented as average values ± standard deviation.

High plasma levels of both SAA (110 mg/L) and SAP (50 mg/L) were found in AKU patient as well as high plasma levels of proinflammatory cytokines (Figure 9).

## 4. Discussion

AKU patients have a higher prevalence of aortic valve disease than normal population [5]. Frequent coronary artery calcification has been reported and AKU patients often require coronary artery bypass grafting [1]. Recently, we reported that AKU is a novel kind of secondary amyloidosis [16] and also assessed the presence of AA amyloid in AKU heart [20]. Our present investigation confirmed the existence of AKU-linked secondary amyloidosis involving the cardiac district. The striking colocalization of pigment and amyloid suggested that HGA might be involved in amyloid deposition in the heart. It is tempting to speculate that this amyloid presence may represent a cardiac manifestation of alkaptonuria, secondary to ochronosis of the cardiovascular system. SAA-amyloidosis of the AKU heart valve thus appears as a novel type of alteration.

SAA-amyloidosis complicating chronic inflammatory diseases involves the heart only in about 2% of cases with systemic AA-amyloidosis and amyloid substance within stenotic aortic valves has been cited to promote mineralization [24]. The present case report is therefore a new rare case of SAA-amyloidosis in the heart.

There is theoretical concern that ochronosis of the fibrous skeleton of the heart could lead to abnormalities of atrioventricular conduction [1]. The presence of amyloid associated with ochronotic pigment reported here may concur in the worsening of cardiac valve impairment. It is conceivable that the development of disruptive tissue lesions is triggered by ochronotic pigment and amyloid deposition and that these altered conditions may cause at least some of the clinical symptoms associated with these lesions. Moreover, we found that in AKU aortic valve amyloid colocalized not only with ochronosis but also with the presence of lymphocytes/macrophages, oxidative damage, calcification, and areas characterized by cell degeneration and cell death.

Analogously to other AKU tissues [9, 12, 25], accumulation of oxidised HGA (BQA) may induce extensive tissue degradation and oxidative stress in the valve. In this study, intraleaflet pigmentation was positively correlated with 4-HNE staining, a known marker of oxidative tissue damage. LPO detected in AKU valve was probably induced by HGA and its oxidised products, as we already reported in other AKU biological systems [14]. We also found macrophages uniformly present in LPO-rich ochronotic areas and markedly abundant in the surrounding connective tissue cells. In fact, oxidized lipids attract inflammatory cells, the predominant cell type in aortic valve lesions, like T lymphocytes and macrophages [26, 27]. Oxidized lipids are highly cytotoxic for most cells [28, 29] and their components have proinflammatory and growth stimulatory properties [27, 30, 31]. It is thus possible that products generated by HGA/BQA-induced lipid oxidation are involved in the inflammatory process present in the AKU valves. We previously reported that HGA induces SAA and proinflammatory cytokines production and release accompanied by oxidative stress (LPO), amyloid and ochronotic pigment production, and cell death in an AKU cell model [13, 14, 18, 19].

Activated T lymphocytes within the subendothelium and fibrosa release cytokines, such as tumor necrosis factor-*α* (TNF-*α*), a pleiotropic cytokine secreted mainly from macrophages, able to contribute to the calcification process of the aortic valve [21]. We found that AKU patient had high plasma levels of SAA (110 mg/mL) and proinflammatory cytokines levels (Figure 9). In particular, transforming growth factor-*β*1, interleukin-1*β*, and TNF-*α*, involved in extracellular matrix formation, remodeling, and local calcification [10, 26, 32, 33] were overexpressed.

SAA is implicated in rheumatoid inflammatory processes through stimulating chondrocytes to produce IL-6 that in turn is recognized as the main inducer of most acute phase proteins, including SAA. Therefore, SAA-induced IL-6 production may enhance the sustained SAA production in an autocrine manner that perpetuates the rheumatoid inflammation. In a recent work, we showed that chondrocytes extracted from AKU patients cartilage produce high levels of IL-8 and IL-6, suggesting that these interleukins could play a role in AKU progression [17].

Mechanical stress, one of the typical functional features of heart valve, may be a linker between amyloid and ochronotic pigment copresence, since it has been reported to be responsible for both amyloid deposition [20, 24, 25] and ochronotic pigment deposition in AKU [6, 9]. Mechanical stress in heart valves has been positively related to oxidation of lipoproteins inducing calcification in pathologies [20, 25, 28, 34].

In addition,* in vitro* experiments have shown that oxidized lipids may be active contributors to inflammation and the mineralization of vascular cells [27].

The presence of amyloid in AKU valve may represent yet another example of amyloid deposition as an integral process in the development of, or in response to, degenerative tissue change due to the aggressive action of ochronotic pigment. Therefore, it is strongly suggested that, in AKU, this condition is a sequel of local complication of progressive destruction and scarring of the valvular connective tissue.

With regard to the pathogenesis of AKU heart amyloidosis, three possible factors may contribute: (i) high levels of circulating plasma SAA as amyloid precursor, as we previously reported to be a common condition in AKU patients [16]; (ii) an increased permeability of the damaged valvular surface; and (iii) the local presence, within the altered ground substance of the valvular connective tissue, of the ochronotic pigment having close structural connection with SAA-amyloid, as we reported in the present work and previously in joint tissue [16]. This condition may be worsened by the local* HGD* expression in heart, reported here. This latter may explain why, although valve is poorly vascularised, ochronosis is relevant in such type of tissue and why aortic valve stenosis and/or regurgitation is the most significant AKU clinical cardiovascular manifestation.

One of the major findings of our study is that heterotopic ossification, an active process of abnormal tissue repair, occurred in end-stage AKU valvular heart disease. AKU is an inflammatory disease [9, 11–18, 23]. The results from the present study are consistent with cardiac valve calcification and ossification also being an inflammatory process. Moreover, macrophages and lymphocytes accumulate in areas of dystrophic calcification and ossification, as we observed in AKU valve. Furthermore, proinflammatory molecules, such as TNF-*α*, released from lymphocytes, can contribute to mineralization and ossification of the valve. Based on this knowledge, and on results of studies showing the role of macrophage-derived inflammatory cytokines (IL-1*β*, TNF-*α*, IL-6, and TGF-*β*) in promoting osteogenic differentiation of vascular smooth muscle cells, we can obtain a detailed picture of the pathological process at molecular level and suggest an inflammation-dependent calcification.

## 5. Conclusion

It appears that there are at least four major events going on within the AKU aortic valve calcification that converge to enhance disease progression: (i) ochronotic pigment production; (ii) amyloid deposition; (iii) inflammation; and (iv) endochondral ossification. We can assume that a sequence of events happen, starting an early phase from local injury to the endothelium due to ochronotic pigment causing its damage. This aggressive burden may promote a deleterious inflammatory process at the valvular fibrosa, followed by inflammatory cellular infiltration that was evident in our results. This would be associated with release of cytokines within the subendothelium and fibrosa resulting in increased collagen production by the fibroblasts within the connective tissue and finally resulting in thickening of the valvular leaflets. In this phase, activated macrophages infiltrate the valvular tissue and release proinflammatory and proosteogenic cytokines. In the propagation phase, further compositional changes may take place due to the presence of activated macrophages, resulting in disruption of collagen and elastin fibres, which together with matrix vesicles and apoptotic bodies may provide a core for* in situ *calcification. And finally, the end-stage characterized by heterotopic bone formation, where the intact well-functioning aortic valve is replaced by pronounced calcification.

According to our postulation, AKU calcific stenosis is a multifactorial situation involving a sequence of events. Hence, any treatment strategy should take into consideration that AKU calcific aortic stenosis pathogenesis bares three different phases. Thereby, it would require a multitarget mechanism for its modulation being directed according to the stage. Early stages might involve the elimination of the cause of endothelial damage like circulating SAA and possibly HGA. Also it would involve inhibition of inflammatory cellular infiltration. Later, the role of therapies that interfere with cellular oxidative stress would come as well as those having role in affecting calcification.
